# Bioinformatic analysis and experimental identification of blood biomarkers for chronic nonunion

**DOI:** 10.1186/s13018-020-01735-1

**Published:** 2020-06-05

**Authors:** Jingwei Wu, Limin Liu, Huaijian Hu, Zhihua Gao, Shibao Lu

**Affiliations:** Department of Orthopedics, Xuanwu Hospital, Capital Medical University, Beijing, 100053 People’s Republic of China

**Keywords:** Fracture, Nonunion, Biomarker, Dipsaci Radix (DR)

## Abstract

**Background:**

Incomplete fracture healing may lead to chronic nonunion; thus, determining fracture healing is the primary issue in the clinical treatment. However, there are no validated early diagnostic biomarkers for assessing chronic nonunion. In this study, bioinformatics analysis combined with an experimental verification strategy was used to identify blood biomarkers for chronic nonunion.

**Methods:**

First, differentially expressed genes in chronic nonunion were identified by microarray data analysis. Second, Dipsaci Radix (DR), a traditional Chinese medicine for fracture treatment, was used to screen the drug target genes. Third, the drug-disease network was determined, and biomarker genes were obtained. Finally, the potential blood biomarkers were verified by ELISA and qPCR methods.

**Results:**

Fifty-five patients with open long bone fractures (39 healed and 16 nonunion) were enrolled in this study, and urgent surgical debridement and the severity of soft tissue injury had a significant effect on the prognosis of fracture. After the systems pharmacology analysis, six genes, including QPCT, CA1, LDHB, MMP9, UGCG, and HCAR2, were chosen for experimental validation. We found that all six genes in peripheral blood mononuclear cells (PBMCs) and serum were differentially expressed after injury, and five genes (QPCT, CA1, MMP9, UGCG, and HCAR2) were significantly lower in nonunion patients. Further, CA1, MMP9, and QPCT were markedly increased after DR treatment.

**Conclusion:**

CA1, MMP9, and QPCT are biomarkers of nonunion patients and DR treatment targets. However, HCAR2 and UGCG are biomarkers of nonunion patients but not DR treatment targets. Therefore, our findings may provide valuable information for nonunion diagnosis and DR treatment.

**Trial registration:**

ISRCTN, ISRCTN13271153. Registered 05 April 2020—Retrospectively registered.

## Introduction

Fracture healing is a complex process and is dependent on multiple factors [1]. Limited fracture healing can occur in 5–10% of fracture cases [2], resulting in chronic nonunion and functional disability, which can have a devastating impact on the patient’s quality of life [3]. The determination of nonunion is essential for fracture diagnosis and subsequent treatment, but there is a lack of objective tools to assess fracture healing, making nonunion as an uncertain outcome [4]. Therefore, establishing a new diagnosis method is vital for chronic nonunion diagnosis and treatment. Undoubtedly, the circulating biomarkers of bone fracture healing are gaining popularity as possible early predictors of chronic nonunion [5]. However, there are currently no valid biomarkers for chronic nonunion diagnosis in the blood.

In fracture healing, monocytes are involved in angiogenesis [6] and differentiate into osteoclasts [7]. Many bone formation-related proteins were changed in human peripheral blood mononuclear cells (PBMCs) after bone fracture [8], which indicated that the altered genes of PBMCs might be early predictors of chronic nonunion. To investigate this possibility, the differentially expressed genes were analyzed using microarray datasets, and potential biomarkers were predicted by a systems pharmacology approach.

Dipsaci Radix (DR) is derived from Dipsacus asperoides, a classical, traditional Chinese medicine with a long history of safe use for the treatment of bone fractures [9, 10]. DR can also be used for the treatment of lower back pain and traumatic hematoma [11]. Moreover, growing evidence indicates that DR could improve osteoporosis by increasing bone density and bone strength [12, 13]. In addition, many studies have confirmed that DR can effectively reestablish the dynamic balance between bone formation and bone resorption [12]. However, no research has examined the potential of DR in the treatment of chronic nonunion.

In this study, bioinformatics analysis methods, including microarray data analysis and an integrated systems pharmacology approach, were used to predict the potential biomarkers of chronic nonunion and mechanisms of DR treatment. First, the differentially expressed chronic nonunion genes in PBMCs were identified using microarray datasets. Second, the potential active ingredients of DR were used to screen the possible target genes. Third, the DR target genes that were also potential biomarkers of chronic nonunion were obtained. Finally, the predicted biomarkers of chronic nonunion were verified by experimental methods, such as enzyme-linked immunosorbent assay (ELISA) and quantitative polymerase chain reaction (qPCR), with blood samples from healed and nonunion patients. Our study provides a more specific and effective way to investigate blood biomarkers for chronic nonunion and provides new insight into the mechanisms of DR in the treatment of chronic nonunion.

## Materials and methods

### Microarray data and difference analysis

Microarray datasets GSE93138, GSE93213, and GSE93215 were downloaded from the Gene Expression Omnibus (GEO) database [14] and collected using the GPL6244 platform (Affymetrix Human Gene 1.0 ST Array). These microarray datasets form the study “Biomarker Identification in Fracture Healing.” This study aimed to identify potential peripheral blood biomarkers for normal healers, slow healers, and nonunion patients [15], which is consistent with the design of our study. Difference analysis was performed by R script using the limma (linear models for microarray analysis) R package, with *p* < 0.05 and |logFC| > 1 as cutoff values for screening differentially expressed genes (DEGs). The DEGs of each comparison group are shown as volcano plots. The interaction of the DEG sets was obtained by Venn diagram.

### DR ingredient profiling and drug target gene identification

The bioactive ingredients of DR were collected from the Traditional Chinese Medicine Systems Pharmacology (TCMSP) database [16]. We set drug-likeness (DL) ≥ 0.18 and oral bioavailability (OB) ≥ 30% as the threshold for bioactive ingredients. The structures of the ingredients were downloaded from the PubChem database [17]. Afterward, the target genes corresponding to the compounds were screened from the PharmMapper database [18] and the Swiss Target Prediction database [19]. The details of the data are shown in a previous study [20].

### Network establishment

The STRING database [21] was used to analyze the protein-protein interactions (PPIs) of the DEGs of healed and nonunion patients, and the hub genes were counted by R script. The interactions of the DEGs and DR target genes were obtained by Venn diagram. Based on the potential target genes, the Cytoscape software (version: 3.7.2) was used to construct the drug-disease network. The GeneCards database was used to analyze the function of the potential biomarkers.

### Gene ontology enrichment analysis

The GO enrichment analysis was performed using the Database for Annotation, Visualization, and Integrated Discovery (DAVID) [22], which is an integrated online biological knowledge base and analytical tool. In our study, the target genes were mapped into DAVID to identify the biological processes, molecular function, and cellular components of the predicted target genes involved.

### Patients and treatment

Patients admitted to the Department of Orthopedics of Xuanwu Hospital from August 2018 to July 2019 were enrolled in this study. Participants were skeletally mature, and all were diagnosed with only one open fracture of a long bone, including the humerus, radius/ulna, femur, and tibia/fibula. Exclusion criteria included patients who had a severe head injury, renal insufficiency, liver disease, systemic inflammation (CRP > 0.5 mg/dL), and osteoporosis. Patients whose initial surgical debridement occurred within 8 h and those whose surgical debridement occurred after 8 h were treated with internal fixation according to the type of fracture by well-trained orthopedic surgeons. Ultimately, 55 participants were enrolled in the study and set as the acute injury (AI) group. Then, the AI group was categorized into healed and nonunion groups according to the amendment from the Food and Drug Administration (FDA) on the diagnostic criteria for nonunion. The FDA defines nonunion as a fractured bone that has not completely healed within 9 months of the initial injury and without signs of healing for at least 3 months [23]. In this study, we chose 9 months as the time point to define nonunion. A total of 39 healed patients and 16 nonunion patients were selected in this study, and the clinical characteristics of the patients are shown in Table 1. Moreover, 30 healthy participants were included as the healthy control (HC) group, and no participant in the HC group experienced any fracture. Some of the bone fracture patients were taking DR (21 of the healed patients and 7 of the nonunion patients), and all the patients were followed up every 3 months until 12 months. All procedures performed in the study were approved by the Ethics Committee of Xuanwu Hospital, Capital Medical University (Beijing, China), and informed consent was obtained from all individuals involved in this study or their guardians.

**Table 1:** Characteristics of 55 patients with open fractures of long bone

Characteristics	Healed (*n* = 39)	Nonunion (*n* = 16)	*p* value*
Age (mean ± SD)	38.28 ± 15.68	41.78 ± 16.04	0.46
Gender (M/F)	21/18	9/7	0.87
Smoker	19	11	0.31
Comorbidities	9	5	0.53
Traffic accident	27	13	0.36
Multi-trauma	23	11	0.50
Gustilo grade (III)	3	5	0.02
Initial surgical debridement within 8 h	27	6	0.03
DR treatment	21	7	0.50

^*^Data analyzed by Student’s *t* test or chi-square *χ*^*2*^ test

### Serum collection and ELISA

Whole blood samples were taken from all participants and centrifuged for 5 min at 4000×*g*, and serum was isolated and stored at − 80 °C. The serum concentrations of the biomarkers were measured using separate ELISA kits (Mlbio, Shanghai, China) according to the manufacturer’s instructions.

### PBMC isolation and qPCR analysis

Five milliliters of peripheral blood was taken from all participants. Ficoll-Paque density gradient centrifugation was used to isolate the PBMCs, and the cells were washed with 4 °C phosphate-buffered saline (PBS). Then, the RNeasy Mini Kit (Qiagen, Germany) was used to extract the total RNA, which was reverse transcribed by the ThermoScript™ RT-PCR System (Invitrogen, Carlsbad, CA, USA). mRNA expression was detected by a Talent qPCR kit (TIANGEN, Beijing, China). The primers used in this study are shown in Table 2.

**Table 2:** The primer sequences for real-time PCR

Gene	Forward primer	Reverse primer
CA1	5′-GCTACAGGCTCTTTCAGTT-3′	5′-GACTCCATCCACTGTATGTT-3′
MMP9	5′-TGTACCGCTATGGTTACACTCG-3′	5′-GGCAGGGACAGTTGCTTCT-3′
QPCT	5′-TCTTCGGCAAATTGCAGAAGG-3′	5′-CGGGTATCGCTCTATCAGCA-3′
GAPDH	5′-ACCACAGTCCATGCCATCAC-3′	5′-TCCACCACCCTGTTGCTGTA-3′

### Statistical analysis

Statistical analysis was performed by the GraphPad Prism software version 7.0 (CA, USA). All data are displayed as the mean ± SEM. The comparisons between the two groups were analyzed using two-tailed unpaired Student’s *t* tests or one-way or two-way ANOVA. Categorical data were analyzed using the chi-squared (*χ*^*2*^*)* test. Values of *p* < 0.05 were considered significant.

## Results

### The clinical characteristics of patients with open fractures of long bones

The clinical characteristics of the nonunion group and the healed group are shown in Table 1. There were no significant differences in age, gender, or DR treatment (*p* > 0.05). Eleven of nonunion patients were smokers, compared to 19 healed patients (*p* > 0.05). The majority of fractures occurred in healthy patients, and 5 nonunion patients had comorbidities, compared to 9 healed patients (*p* > 0.05). Most fractures were the result of a traffic accident, and 13 of the nonunion fractures were caused by traffic injury, compared to 27 of the healed fractures (*p* > 0.05). The larger proportion of injuries resulted from multi-trauma, and 11 of the nonunion fractures were from multi-trauma, compared to 23 of the healed fractures (*p* > 0.05). The severity of the soft tissue injury was assessed using the Gustilo classification, and 5 nonunion fractures were Gustilo grade III, compared to 3 healed fractures (*p* < 0.05). The initial surgical debridement times of 6 nonunion and 27 healed fractures were within 8 h, and 10 nonunion and 12 healed fractures received the initial surgical debridement after 8 h (*p* < 0.05). The results above indicate that urgent surgical debridement and the severity of soft tissue injury have a significant effect on the nonunion of open long bone fractures.

### Identification of differentially expressed genes in the PBMCs of healed and nonunion patients

**Fig. 1:**
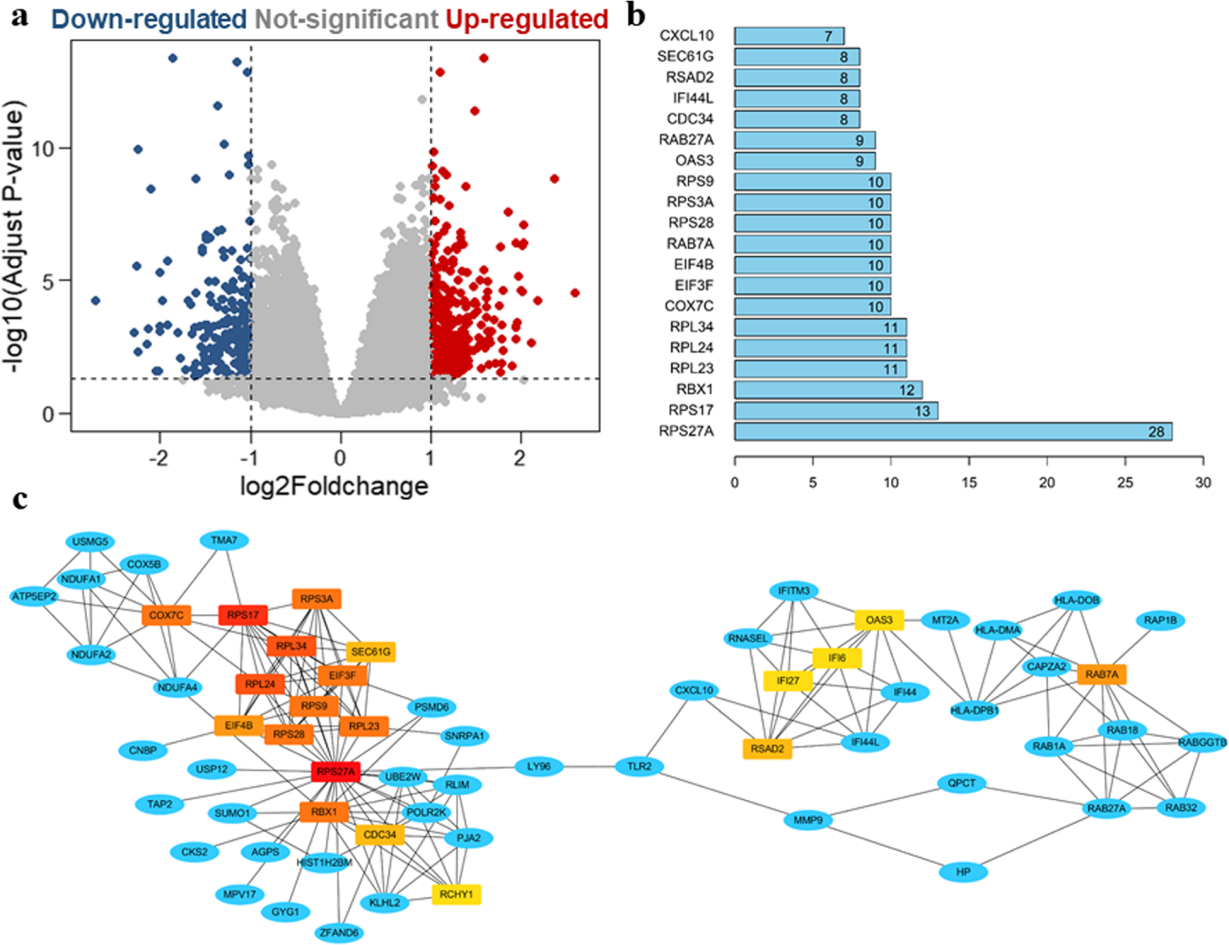
Bioinformatics analysis of differentially expressed genes in PBMCs of healed and nonunion patients. **a** Volcano plot of differential gene expression in PBMCs between healed and nonunion patients. **b** The interaction network of the differentially expressed genes. Bar plot of the number of hub gene links. **c** The protein-protein interaction (PPI) network of the differentially expressed genes; the red and yellow nodes are the hub genes in the network. A deeper red color indicates more connections

Three PBMC gene expression datasets (GSE93138, GSE93213, and GSE93215) of healed and nonunion patients were downloaded from the GEO database. Gene comparison analysis was performed on these two groups, and there were a total of 258 differentially expressed genes between healed and nonunion patients (Fig. 1a). To explore the relationship between these 258 genes, protein-protein interaction (PPI) analysis was performed using the STRING database. A network was generated, and hub genes were analyzed using the Cytoscape software. The top 20 hub genes in the network were marked in red and yellow (the deeper shades of red indicated greater connections of nodes), and other genes connected to hub genes were presented as blue nodes (Fig. 1b). A bar plot of the number of hub gene links is shown in Fig. 1b, and we found that hub genes, such as RPS27A, RPS17, and RBX1, may play a critical role in the biological activity of chronic nonunion.

### Prediction of gene function using enrichment analysis for the differentially expressed genes

**Fig. 2:**
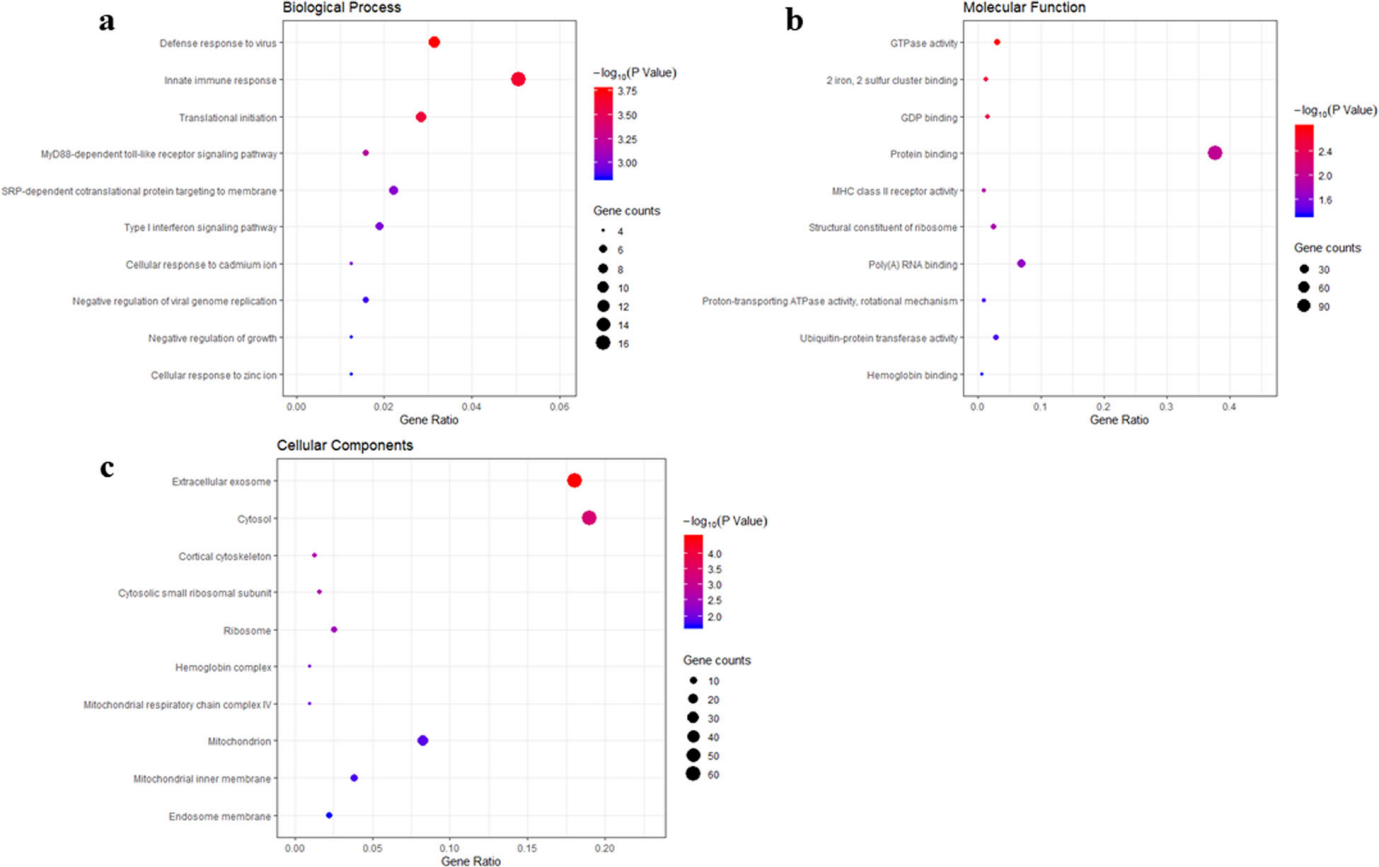
GO enrichment analysis of the differentially expressed genes. **a** Plot of enriched biological processes. **b** Plot of enriched molecular functions. **c** Plot of enriched cellular components. The number of genes enriched in each GO term is shown as the circle size, and the *p* values are shown as different colors

To gain a comprehensive understanding of the differentially expressed genes, we used DAVID to perform GO enrichment analysis. A variety of GO terms were enriched, including 43 biological processes, 21 cellular components, and 17 molecular functions. The top 10 GO terms are shown in Fig. 2. The biological processes (such as innate immune response and translational initiation, Fig. 2a), molecular function (such as GTPase activity and protein binding, Fig. 2b), and cellular components (such as extracellular exosome and cytosol, Fig. 2c) may be involved in the biological activity in chronic nonunion.

### Prediction of nonunion-related biomarkers by a systems pharmacology approach

**Fig. 3:**
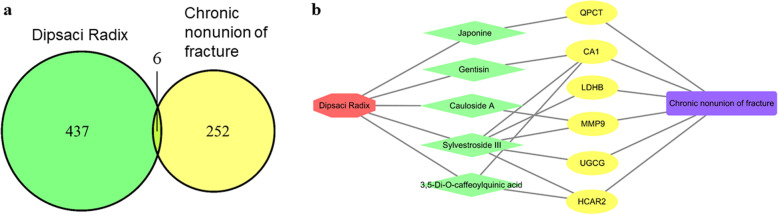
Overlap and network of Dipsaci Radix (DR) target genes and nonunion differentially expressed genes. **a** Venn diagram of DR target genes and nonunion differentially expressed genes. **b** The network of DR, DR components, nonunion, and six potential gene targets. Green nodes represent DR components, and yellow nodes represent gene targets

In this study, we chose Dipsaci Radix (DR), a classical medicine for the treatment of bone fractures, to predict biomarkers for nonunion patients. Ten potential active compounds were retrieved from the TCMSP database, and a total of 443 target genes were obtained using the PharmMapper and Swiss Target Prediction databases. Finally, the interaction of drug target genes and nonunion-related genes was determined using a Venn diagram. As shown in Fig. 3a, we obtained six interacting genes. Moreover, we constructed an interactive network to connect DR, DR compounds, target genes, and nonunion (Fig. 3b). In our study, we found that five active ingredients of DR (Japonine, Gentisin, Gauloside A, Sylvestroside III, and 3,5-Di-O-caffeoylquinic acid) and six target genes (QPCT, CA1, LDHB, MMP9, UGCG, and HCAR2) were related to nonunion. To better understand the function of the potential biomarkers, the GeneCards database was used to find acute phase symptom-related genes. As shown in Table S1, all six biomarkers were related to the acute phase. Five biomarkers were related to multiorgan failure (CA1, LDHB, MMP9, UGCG, and HCAR2) and inflammation (QPCT, CA1, LDHB, MMP9, and HCAR2), four biomarkers were related to the shock (CA1, LDHB, MMP9, and UGCG), and two were related to acidosis (CA1 and LDHB). Based on the systems pharmacology analysis of these predictions and the results of the network analysis, the six potential biomarkers of nonunion were validated using ELISA and qPCR methods.

### Validation of potential biomarkers in serum by ELISA

**Fig. 4:**
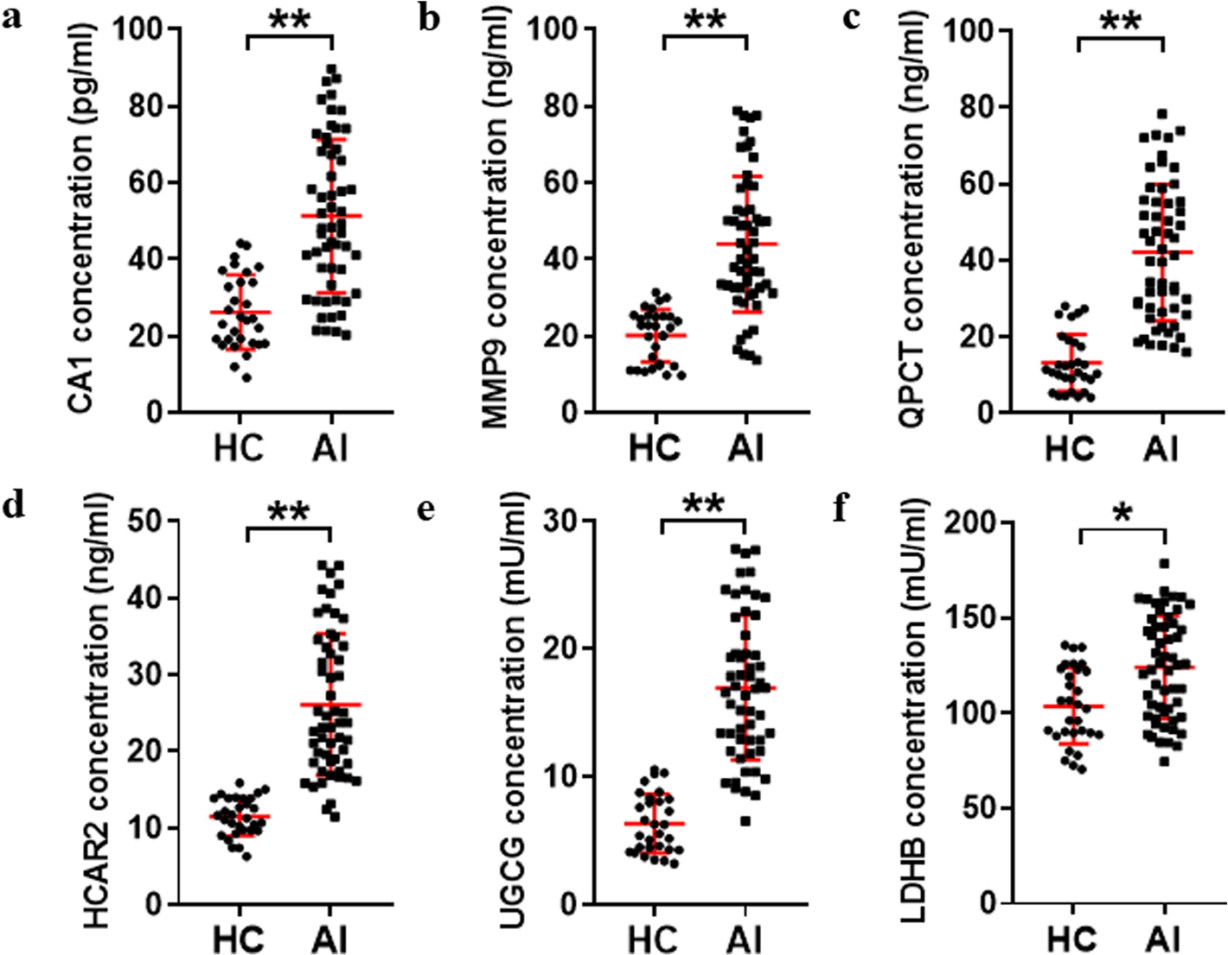
Validation of the six potential biomarkers between healthy control (HC) and acute injury (AI) patients by ELISA. The concentrations of CA1 (**a**), MMP9 (**b**), QPCT (**c**), HCAR2 (**d**), UGCG (**e**), and LDHB (**f**) in serum samples from the HC (*n* = 30) and AI groups (*n* = 55). The error bars represent means ± SEM. ^*^p < 0.05, ^**^p < 0.01, vs. HC group

**Fig. 5:**
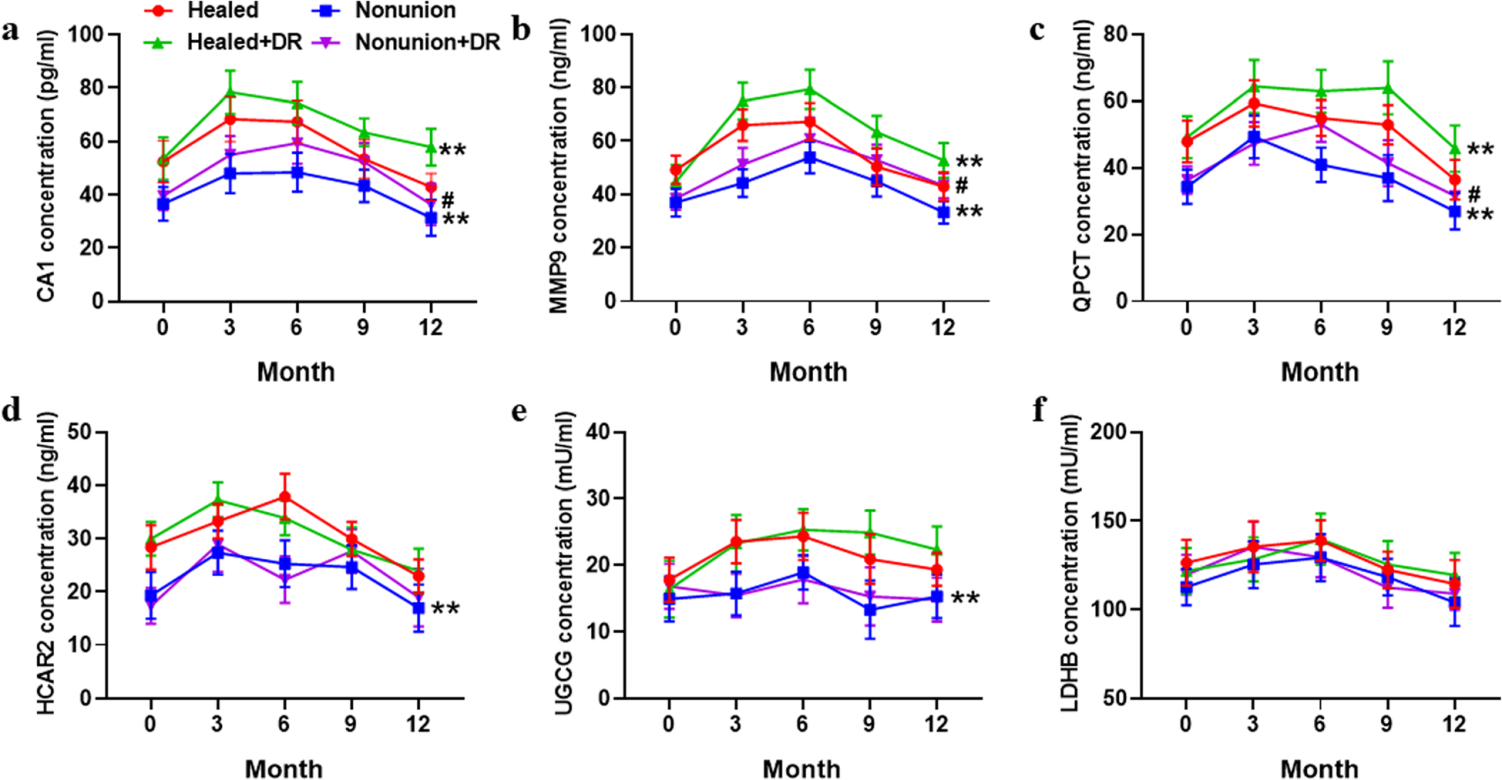
Changes in the six biomarkers among different groups of patients at different times by ELISA. Concentration of CA1 (**a**), MMP9 (**b**), QPCT (**c**), HCAR2 (**d**), UGCG (**e**), and LDHB (**f**) in serum samples of the healed (*n* = 18), nonunion (*n* = 9), healed+DR (*n* = 21), and nonunion+DR (*n* = 7) groups at time points 0, 3, 6, 9, and 12 months. Data are represent as the means ± SEM. ^**^p < 0.01 vs. healed group, ^#^*p* < 0.05 vs. nonunion group

ELISA was performed to test the six potential biomarkers in serum samples from healthy control (HC) and acute injury (AI) patients. As shown in Fig. 4, the serum concentrations of all six potential biomarkers were increased considerably in the AI group (*p* < 0.05). The results showed that after bone fracture, the six genes were all significantly increased, which may be closely related to the fracture healing process. However, the serum concentrations of all six potential biomarkers were not significantly different between fracture-only and multi-trauma patients (Fig. S1). To further observe the changes in the six biomarkers at different times, we divided the AI patients into four groups: healed, nonunion, healed+DR, and nonunion+DR, and then detected the potential biomarkers at the 0, 3-, 6-, 9-, and 12-month time points. We found that the serum concentrations of CA1, MMP9, QPCT, HCAR2, and UGCG were considerably decreased in the nonunion groups (*p* < 0.01, Fig. 5a–e), but there were no significant changes in LDHB (Fig. 5f). DR treatment significantly increased serum concentrations of CA1, MMP9, and QPCT in both the healed and nonunion groups (*p* < 0.05, Fig. 5a–c), but the HCAR2, UGCG, and LDHB concentrations were not changed (Fig. 5d–f). The six potential biomarkers were all increased after acute injury and gradually decreased after 6 months. The results above indicated that CA1, MMP9, QPCT, HCAR2, and UGCG could be biomarkers to identify nonunion patients, and the treatment of DR may target CA1, MMP9, and QPCT; accelerate fracture healing; and minimize delayed healing and nonunion.

### Further validation of potential biomarkers in PBMCs by qPCR analysis

**Fig. 6:**
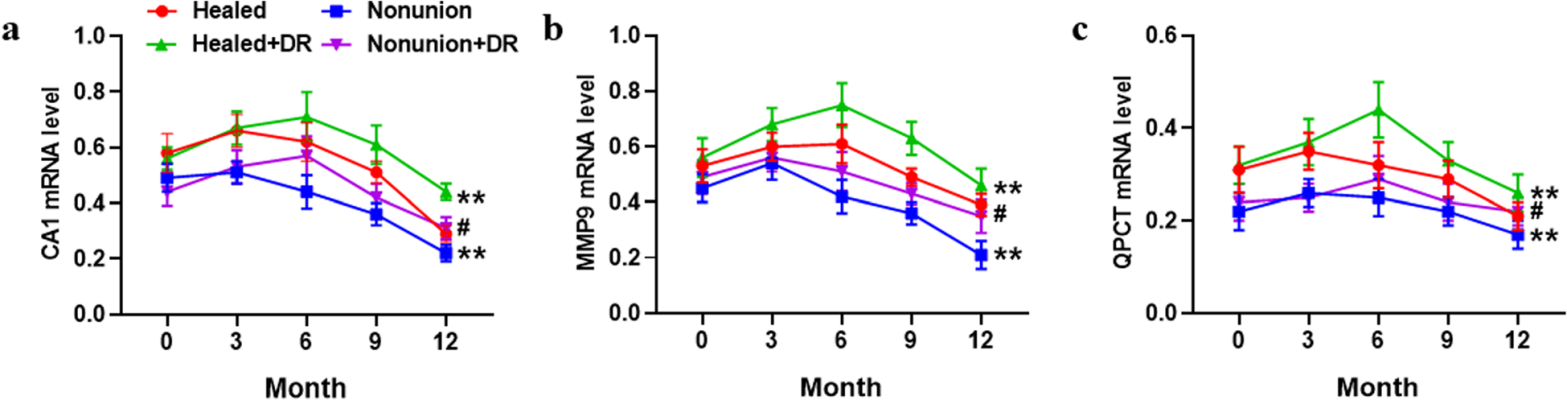
Further observation of the three biomarkers among different groups of patients by qPCR analysis. The mRNA expression levels of CA1 (**a**), MMP9 (**b**), and QPCT (**c**) in PBMC samples from the healed (*n* = 18), nonunion (*n* = 9), healed+DR (*n* = 21), and nonunion+DR (*n* = 7) groups at time points 0, 3, 6, 9, and 12 months. Data were normalized to GAPDH, and values represent the means ± SEM. ^**^p < 0.01 vs. healed group, ^#^*p* < 0.05 vs. nonunion group

To further validate the CA1, MMP9, and QPCT in nonunion patients, we performed qPCR to identify the changes in the three potential biomarkers in PBMCs from different groups of patients. As shown in Fig. 6, the PBMC mRNA expression levels of CA1, MMP9, and QPCT were significantly changed in the nonunion groups (*p* < 0.05). DR treatment significantly increased CA1, MMP9, and QPCT, PBMC mRNA expression levels in both the healed and nonunion groups (*p* < 0.05, Fig. 6a–c), consistent with the ELISA results. The results confirmed that CA1, MMP9, and QPCT could be biomarkers to identify nonunion patients and DR treatment targets.

## Discussion

Fracture healing is a continuous process that greatly depends on the location and type of fracture, the choice of treatment, and other factors related to the host and injury [24]. No standard diagnostic criteria for chronic nonunion has been shown to delay the requirement for treatment, which costs significantly more than those uncomplicated fracture healing [25]. Aside from radiological and clinical examination, serologic markers show promise in predicting the status and quality of fracture healing [26]. The ideal biomarker of bone healing must have the characteristics of accuracy, high sensitivity, specificity, and rapidity, and be inexpensive, and able to predict the outcome of bone nonunion [27]. However, due to the lack of relevant clinical evidence, it is difficult to determine which biomarkers can be reliably used for clinical follow-up and prediction of chronic nonunion [28].

In our study, we predicted the potential blood biomarkers of nonunion through a series of bioinformatics analyses and combined with subsequent experimental verification. Based on this strategy, we predicted 258 differentially expressed genes in PBMCs between healed and nonunion patients. To further understand the function of these genes, PPI analysis and GO enrichment analysis were conducted. We found that hub genes, such as RPS27A, RPS17, and RBX1, may play a critical role in the biological activity in nonunion patients. The innate immune response, GTPase activity, extracellular exosomes, and cytosol may be involved in the altered biological activity in nonunion patients.

After analysis of these 258 genes by a systems pharmacology approach using the DR drug; six genes, including QPCT, CA1, LDHB, MMP9, UGCG, and HCAR2, were chosen for experimental validation by ELISA and qPCR methods. We verified that all six genes were differentially expressed after acute injury, and five genes (QPCT, CA1, MMP9, UGCG, and HCAR2) were significantly lower in nonunion patients. Meanwhile, CA1, MMP9, and QPCT were markedly increased in patients using DR treatment, suggesting that the proteins might serve as potential blood biomarkers for nonunion and may be potential DR treatment targets. It is worth noting that the six potential biomarkers were related to the acute phase, shock, acidosis, multiorgan failure, and inflammation, but the specific changes of the biomarkers in these manifestations require further study.

Matrix metalloproteases (MMPs) are a family of 23 zinc-dependent proteolytic enzymes that can cleave the extracellular matrix (ECM). MMPs play an essential role in tissue regeneration and bone remodeling processes [29]. Previous studies have shown that MMP9 contributes to bone healing processes, which are essential during fracture repair [30, 31]. Furthermore, MMP9 can be considered an early marker of tissue healing; a high level of MMP9 is indicative of the beginning of the bone remodeling process [32]. In this study, we predicted and verified that the level of MMP9 in serum and PBMCs was significantly lower in the nonunion group than in the healed group. Moreover, DR treatment can promote MMP9 expression, which indicates that MMP9 is not only a biomarker of nonunion patients but also a treatment target of DR.

Carbonic anhydrase I (CA1) is a member of the carbonic anhydrase (CA) family, which catalyzes the reversible hydration and dehydration reactions of CO_2_/H_2_CO_3_ [33]. A study has shown that CA1 stimulates calcium deposition and cell calcification, which is an essential step for new bone formation [34]. Moreover, CA1 recruitment of CO_3_^2−^ ions is also regarded as an essential part of bone fracture healing [35]. In the present study, CA1 was significantly changed in the nonunion group compared with the healed group and can also be increased by DR treatment, which indicated that CA1 was a potential biomarker and treatment target of nonunion patients.

Pituitary glutaminyl cyclotransferase (QPCT), also known as pituitary glutaminyl cyclase, can convert active forms of gonadotropin-releasing hormone (GnRH) peptides to protected forms [36]. A previous study reported that the QPCT gene could affect bone mineral density (BMD) among postmenopausal Japanese women [37], which was also verified in the Chinese population [38], and together, these findings indicated that the QPCT gene is one of the osteoporosis susceptibility genes. However, the function of QPCT in bone formation has not been reported. In the present study, QPCT was significantly increased after bone fracture and DR treatment but much lower in the nonunion group than in the healed group, which indicated that QPCT has a function in promoting the remodeling process. Further studies that focus on the roles of QPCT in the diagnosis and treatment of nonunion patients are essential and of great interest.

Hydroxycarboxylic acid receptor 2 (HCAR2) is an endogenous ketone produced by fatty acid oxidation in liver mitochondria during carbohydrate deficiency [39]. HCAR2 has anti-inflammatory and antioxidative properties on immune and epithelial cells [40], but the roles of HCAR2 in bone fracture healing are still unknown. For the first time, we have demonstrated that HCAR2 significantly increases after bone fracture and has the potential to identify the nonunion but it is not the target of DR treatment.

UDP-glucose ceramide glycosyltransferase (UGCG) is the only enzyme responsible for the de novo production of glucosylceramide (GlcCer), which is essential for proper cell function [41]. LDHB is one isoenzyme of human lactate dehydrogenase (LDH), which is a crucial glycolytic enzyme [42] and is distributed in different tissues [43]. Neither UGCG nor LDHB is well-known in the bone repair process. This is the first study presenting a relationship between LDHB, UGCG, and bone fracture. We found that both LDHB and UGCG were increased in bone fracture patients, but only UGCG was significantly lower in the nonunion group, with no significant changes during DR treatment. The results above indicated that UGCG might predict nonunion, but neither UGCG nor LDHB was DR targets.

Our study provides valuable information to investigate blood biomarkers for chronic nonunion and the potential mechanisms of DR in the treatment of bone fracture. Despite the large patient cohort, only a small number of patients could be included in the study due to a lack of objective tools to assess nonunion. Although we attempted to increase the accuracy of the results, there was inevitable interference from other factors, and the predicted genes need to be validated on large-scale blood samples further.

## Conclusion

This bioinformatics analysis combined with the experimental verification strategy provides five potential blood biomarkers for nonunion patients and three DR treatment targets and is the first study to use such an approach for predicting nonunion blood biomarkers. Early diagnosis of chronic nonunion will help clinicians take timely countermeasures to improve bone healing, which will result in better clinical management of patients. In addition, further prospective clinical studies will evaluate the predictive power of these biomarkers for the prognosis of bone fractures. Changes in the potential biomarkers also need to be further studied in nonunion of other fractures in addition to long bone fractures.

## Supplementary information

**Additional file 1: Figure S1.** Validation of the six potential biomarkers between fracture only and multi-trauma patients by ELISA. Concentration of CA1 (A), MMP9 (B), QPCT (C), HCAR2 (D), UGCG (E), and LDHB (F) in serum samples from the fracture only (*n* = 16 in healed group, *n* = 5 in nonunion group) and multi-trauma patients (*n* = 23 in healed group, *n* = 11 in nonunion group). The error bars represent means ± SEM. There has no significant difference between fracture only and multi-trauma patients. **Table S1.** Functional analysis of the six potential blood biomarkers.

## Data Availability

The datasets used and/or analyzed during the current study are available either online or from the corresponding author on reasonable request.

## Abbreviations

DR: Dipsaci Radix
AI: Acute injury
PBMCs: Peripheral blood mononuclear cells
ELISA: Enzyme-linked immunosorbent assay
qPCR: Quantitative polymerase chain reaction
FDA: Food and Drug Administration (FDA)
GEO: Gene Expression Omnibus
DEGs: Differentially expressed genes
TCMSP: Traditional Chinese Medicine Systems Pharmacology
DL: Drug-likeness
OB: Oral bioavailability
PPIs: Protein-protein interactions
DAVID: Database for Annotation, Visualization, and Integrated Discovery
HC: Health control
GO: Gene ontology
MMPs: Matrix metalloproteases
ECM: Extracellular matrix
CA1: Carbonic anhydrase I
QPCT: Pituitary glutaminyl cyclotransferase
BMD: Bone mineral density
HCAR2: Hydroxycarboxylic acid receptor 2
UGCG: UDP-glucose ceramide glycosyltransferase
LDH: Lactate dehydrogenase
