# Supplementary material for: Bioinformatic analysis and experimental identification of blood biomarkers for chronic nonunion
Source: J Orthop Surg Res. 2020 Jun 5;15:208. doi: 10.1186/s13018-020-01735-1 (PMC7275361; doi:10.1186/s13018-020-01735-1)
Supplement: Supplementary file 1 — Additional file 1: Figure S1. Validation of the six potential biomarkers between fracture only and multi-trauma patients by ELISA. Concentration of CA1 (A), MMP9 (B), QPCT (C), HCAR2 (D), UGCG (E), and LDHB (F) in serum samples from the fracture only (n = 16 in healed group, n = 5 in nonunion group) and multi-trauma patients (n = 23 in healed group, n = 11 in nonunion group). The error bars represent means ± SEM. There has no significant difference between fracture only and multi-trauma patients. Table S1. Functional analysis of the six potential blood biomarkers. [file 13018_2020_1735_MOESM1_ESM.docx]

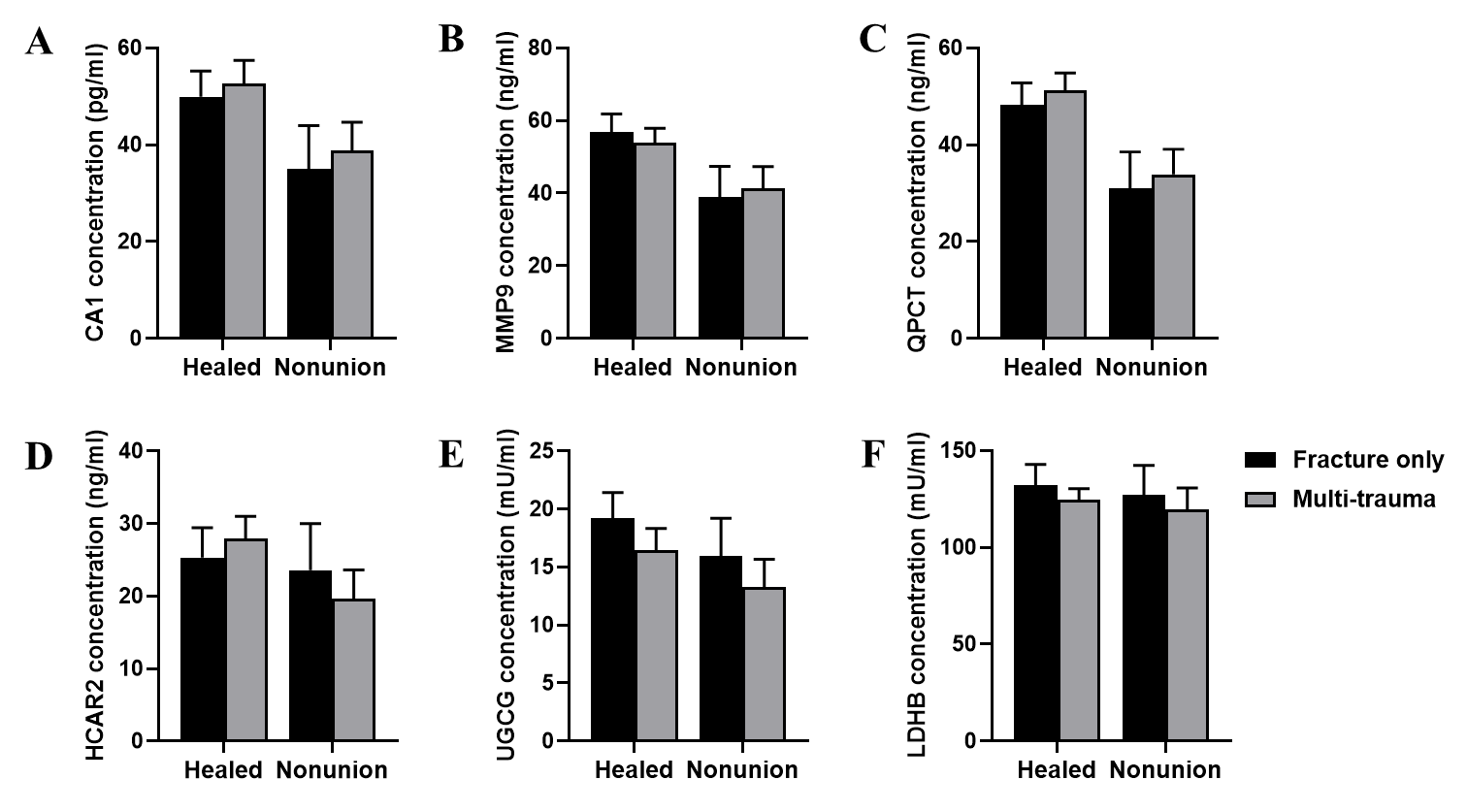


**Fig. S1** Validation of the six potential biomarkers between fracture only and multi-trauma patients by ELISA. Concentration of CA1 (A), MMP9 (B), QPCT (C), HCAR2 (D), UGCG (E), and LDHB (F) in serum samples from the fracture only (*n* = 16 in healed group, *n* = 5 in nonunion group) and multi-trauma patients (*n* = 23 in healed group, *n* = 11 in nonunion group). The error bars represent means ± SEM. There has no significant difference between fracture only and multi-trauma patients.

**Table S1** Functional analysis of the six potential blood biomarkers

| Biomarkers | Acute phase | Shock | Acidosis | Multi-organ failure | Inflammation |
| --- | --- | --- | --- | --- | --- |
| CA1 | √ | √ | √ | √ | √ |
| MMP9 | √ | √ | × | √ | √ |
| QPCT | √ | × | × | × | √ |
| HCAR2 | √ | × | × | √ | √ |
| UGCG | √ | √ | × | √ | × |
| LDHB | √ | √ | √ | √ | √ |
